# Treatment of Pulmonary Tuberculosis by Artificial Pneumothorax

**Published:** 1910-12

**Authors:** S. T. Harris

**Affiliations:** Valdosta, Ga.


### Journal-Record of Medicine

Successor to Atlanta Medical and Surgical Journal, Established 1855, and Southern Medical Record, Established 1870.

OWNED BY THE ATLANTA MEDICAL JOURNAL CO.

Published Monthly

*Official Organ Fulton County Medical Society, State Examining Board, Presbyterian Hospital, Atlanta, Birmingham and Atlantic Railroad Surgeons' Association, Chattahoochee Valley Medical and Surgical Association, Etc.*

EDGAR G. BALLENGER, M. D., Editor.
BERNARD WOLFF, M. D., Supervising Editor.
A. W. STIRLING, M. D., C. M., D. P. H., J. S. HURT, B. Ph., M. D.
GEO. M. NILES, M. D., W. J. LOVE, M. D., (Ala.); Associate Editors.
E. W. ALLEN, Business Manager.

COLLABORATORS

Dr. W. F. WESTMORLAND, General Surgery.
F. W. McRAE, M. D., Abdominal Surgery.
H. F. HARRIS, M. D., Pathology and Bacteriology.
E. B. BLOCK, M. D., Diseases of the Nervous System.
MICHAEL HOKE, M. D., Orthopedic Surgery.
CYRUS W. STRICKLER, M. D., Legal Medicine and Medical Legislation.
E. C. DAVIS, A. B., M. D., Obstetrics.
E. G. JONES, A. B., M. D., Gynecology.
R. T. DORSEY, Jr., B. S. M. D., Medicine.
L. M. GAINES, A. B., M. D., Internal Medicine.
J. N. LeCONTE, M. D., Disease of the Stomach and Intestines.
L. B. CLARKE, M. D., Pediatrics.
EDGAR PAULIN, M. D., Opsonic Medicine.
THEODORE TOEPEL, M. D., Mechano Therapy.
R. R. DALY, M. D., Medical Society.
A. W. STIRLING, M. D., etc., Diseases of the Eye, Ear, Nose and Throat.
BERNARD WOLFF, M. D., Diseases of the Skin.
E. G. BALLENGER, M. D., Diseases of the Genito-Urinary Organs.

VOL. LVI. DECEMBER, 1910 NO. 9

### TREATMENT OF PULMONARY TUBERCULOSIS BY ARTIFICIAL PNEUMOTHORAX.*

By S. T. Harris, of Valdosta, Ga.

Before taking up the subject with which this paper is intended mainly to deal, a preliminary discussion appears necessary of those forms of pneumothorax that are directly the result of disease and that occur under natural conditions; reference will also have to be made to hydrothorax and empyema, as the effect of all of these conditions on pulmonary tuberculosis serve as a basis for the idea that consumption can be treated successfully by artificially collapsing one of the lungs.

Under natural conditions the term *pneumothorax* is used to signify the presence of air in the spaces between the perietal and the visceral layers of the pleurae, and this name also applies to the condition of the pleural cavity when distended by gases other than air. Pathological pneumothorax is usually complicated with hydrothorax or empyema, but when air is artificially introduced into the chest cavity accompanying accumulations of liquid of any kind are exceedingly rare.

*Causes*:—It is interesting to note that natural pneumothorax occurs under a great variety of conditions, and may arise from many pathological states, such, for example, as wounds of the chest, perforation of the diaphragm, or rupture of the lung itself, the last named being a consequence of a tuberculous process in 90% of the cases of pneumothorax not artificially produced. In rare cases perforation may follow pneumonia, abscess, gangrene, haemorrhagic infarction, or hydatid cysts of the lung, and may be a consequence of bronchiectasis, or of suppurating bronchial glands; it may likewise, in rare instances, arise from perforation of the lung in empyema, from the growth of gas-producing organisms in the pleural cavity, and even from violent muscular effort. Very exceptionally it appears to occur from no known pathological cause.

Finally, the condition may be produced by the artificial introduction of gas into the chest cavity. This is known as *artificial pneumothorax*, and is now being employed in the treatment of pulmonary tuberculosis. It is to this procedure that I desire to draw your particular attention.

### Effects of Empyema, Hydrothorax, and Natural Pneumothorax on Tuberculosis of the Lung.

The curative and arresting effect of pleural effusions in phthisis, it is asserted, has been observed for two or three centuries. Osler states that in tuberculous cases where accidental pneumothorax occurs early, the progress of the disease seems to be arrested, and cites a case which was under his care for three or four years.

Hughes, in 1898, reported a case of recovery followed by partial re-expansion of the right lung, in which a pathological pneumothorax had occurred. Later, post-morten findings showed calcified tubercules in the right upper lobe, and a cavity containing chalk-like substance, separated from the surface only by the thickened pleura. This case was complicated with empyema.

Futterer reported a case, in 1896, of an accidental pneumothorax followed by symptomatic recovery. In this instance, he substituted a weak, watery solution of oil of cloves for the air. You will notice here an artificial hydrothorax was substituted for an accidental pneumothorax, which is very suggestive of the harmlessness of hydrothorax in tuberculosis.

It has been estimated that in all cases of empyema occurring in adults that 84% are the result of primary pulmonary tuberculosis. Cabot, Runeberg, and Koenig operated on 134 uncomplicated cases of empyema; four died, seven remained with fistula and were incomplete cures, nine were lost sight of, and the other 114 recovered from their tuberculosis, as well as the empyema. These results speak for themselves, and the explanation of the cure of tuberculosis in them is due to the prolonged compression exerted by the presence of the purulent fluid. Pus would very naturally be absorbed quite slowly in the pleural cavity under such conditions.

Lemke states that the condition of a patient with pulmonary tuberculosis immediately improves on the advent of a simple serous effusion, there is a marked gain in weight and strength, decrease in the number of bacilla in the sputum, and diminution and final disappearance of cough. He suggests that as 65.2% of all instances of so-called indiopathic pleurisy have been estimated as tuberculous, and as patients coming into the hospital with pleurisy were re-admitted months or even years later with phthisis, that these cases were in the beginning tubercular, and that the progress has been arrested for this length of time by the compression due to the effusion. He also states that Murphey had for a number of years refrained from early operating in pleural effusions in order to get the benefit of the compression.

Tidey, in 1896, says, "In health the respiratory capacity of the lungs is commensurate with the semi-circumference of the thorax. The diseased lung may be regarded as decreased in bulk, so far as function is concerned, by the amount of lung involved, and it follows that the best mechanical condition of respiration would be secured by reducing the thoracic cavity in proportion to the reduced bulk of lung. Natural processes of repair tend to secure these conditions, but only lead to complete cicatrization when the disease is of a limited extent. We find then clinically a flattening of the chest wall, dislocation of adjacent organs, or hypertrophy of the opposite lung. Insofar as the inflammatory element in phthisis is concerned, rest and relaxation of the inflamed tissues seem indicated and essential to the healing process." Tidey also says that pleuritic effusions frequently lead to amelioration in phthisis.

Loomis, in 1896, says that "he has seen a number of cases of tuberculosis at the base of the lung, and also advanced cases of ordinary apical tuberculosis, suddenly start to improve and the patient, from being in a desperate condition, became comparatively well. This change followed upon the filling of the chest cavity on the affected side with effusion, the result of tuberculous pleurisy."

From translations by Dr. Lapham, I gather the following data:

Forlanini says, "It has frequently been noted in practice that a pneumothorax or a pyo-pneumothorax spontaneously developed exercises a rapid and remarkable improvement upon the course of tuberculosis." He cites a case where spontaneous pneumothorax, due to perforation of the pleura by a tuberculous process, at once produced a surprising amelioration of all the symptoms. The fever, which had been very high, fell, and there followed a marked improvement of the general health. This was followed by pleuritic effusion, which was withdrawn many times, and finally became purulent. He evacuated the empyema as fast as it was formed, and substituted each time a certain amount of sterile nitrogen, finally obtaining a complete immobilization of the left lung, which condition was maintained. The empyema had been entirely replaced by a pneumothorax. He says the course of this case showed the curative influence of pneumothorax empyema. In this instance three operations sufficed to cure the effusion. He cites another case of a physician, where spontaneous pneumothorax developed an empyema. Such a rapid and remarkable improvement followed that the patient considered himself well, and for some months resumed his ordinary life.

Spath, of Wurtemburg, published a case in 1898, where the base of the right lung had been compressed by an effusion, and the tissues thus protected were entirely normal, all the rest of both lungs being riddled with miliary tubercles. Schmorl published four similar cases in which pleural effusions had protected the compressed tissues from the tubercular process.

These illustrations might be multiplied, but, believing that sufficient evidence has been presented to justify the theory and practice of artificial pneumothorax, I will leave the subject of pathological, or accidental, compression of the lungs, and its effects, and take up a consideration of the histological changes resulting from compression in both the normal and pathological pulmonary tissues.

### History of Artificial Pneumothorax.

The theory of the treatment of pulmonary disease by obtaining rest for the lungs in some manner is now quite old.

In 1880 Forlanini advocated the principle of lung compression by nitrogen gas in the treatment of pulmonary tuberculosis, and is at present the highest authority on this method of dealing with phthisis. Forlanini has done quite a number of successful operations.

In 1898 Murphey, of Chicago, reported five cases in which sterilized nitrogen had been employed to collapse tuberculous lungs with excellent results. Murphey operated on one case this year, which I had the pleasure of observing very closely, this being a case of Dr. Mary E. Lapham, of Highlands Sanitorium, Highlands, N. C.

In 1899 the late Dr. A. F. Lemke, of Chicago reported in detail 53 cases, and stated that he had done about 40 others.

Orlandi, director of the hospital in Arezzo, and Fontana, director of the Piacenza Hospital, and Dr. C. P. Thomas, of Spokane, Wash., have all done some work along this line.

### Results of Artificial Pneumothorax on the Lung Tissue.

Osler says, "In an extensive effusion which reaches to the clavicle, the entire lung will be found lying close to the spine, dark and airless, or even bloodless, i. e., carnified.

Lemke quotes the following:

Rokitansky, in 1861, observed that after long continued compression of the lung a certain degree of fibrosis developed in it. According to Cornil and Ranvier, the changes under such circumstances consist principally of an edema, and an exfoliation of epithelial cells.

Dunin quotes Herz as stating that it had not been proven that the alveoli are permanently obliterated by adhesions to their walls. Futterer stated that in a collapsed lung edema and hyperemia were present and exercised a beneficial influence.

In an autopsy published by Lemke, he found the result of compression in the lung due to artificial pneumothorax to be that linear scars had been formed marking the sites of obliterated cavities, with an extreme degree of fibrosis. There were large and small caseous masses, but all surrounded by thick walls of scar tissue, and fine bundles of connective tissue—fibers were seen to pass into the caseous areas, and even in the very centres of some of these foci fine fibrils were visible.

A case was reported by Forlanini which had been treated by artificial pneumothorax for two years, and which had maintained itself for two years longer, before the patient succumbed to a pneumonia of the right lung. There was anatomical recovery of a severe phthisis of the left lung, which was reduced to a solid mass, cicatricized and strongly adherent to the thoracic walls. Microscopically, this solid mass was composed of more or less compact bands of connective tissue, irregularly distributed and interwoven, and from the midst of this stretched out through the compressed lung, which was deformed, and here and there infiltrated and carnified. All destructive lesions had become cicatricized. There was absence of any kind of pneumonia or caseous material. In a word, every anatomico-pathological process characteristic to tuberculosis had disappeared. This last case seems to typify the ideal result which is obtained from artificial pneumothorax,—a cure, positive and complete.

Symptoms Following Artificial Pneumothorax.

It would appear at first sight that the complete stoppage of the function of one lung would cause most serious subjective inconvenience, but such is not the case. hardly more so than the loss of one eye would cause a cessation of sight. The physiological process of respiration is carried on with very little impairment by the good lung.

Lemke quotes: "Neil and Thomas in an experimental study of hydrothorax and pneumothorax demonstrated that in closed pneumothorax, and in hydrothorax with movable fluid, the minute volume of respiration is increased, and there is an increase in the excretion of carbon dioxide." Sakur in an experimental study of pneumothorax demonstrated by careful measurements that after one lung is collapsed by a pneumothorax the other respires as much as did both together. The symptoms of artificial pneumothorax are usually so slight as to cause very little subjective effect. This fact is well shown in occasional cases of long standing phthisis, where the natural pneumothorax is only discovered postmorten. West says that even in healthy subjects this latent pneumothorax may occasionally occur. On the other hand, dyspnoea of varying grades may follow artificial pneumothorax, which, however, is usually of a transient type and leaves no bad after effects. This condition of dyspnoea has usually been the result of the rapid production of a massive pneumothorax, or possibly to the expansion of air or gases due to the bodily heat after their introduction into the pleural cavities. To safeguard against this condition I suggest the use of small multiple injections, and also that the air or gases be injected at or slightly above the internal bodily heat; this latter practice would, in my opinion, also to a great degree obviate pleuratic shock, which is one of the most distressing and dangerous results of pneumothorax. Pain, which occurs not infrequently, is usually of short duration and minor importance in artificial pneumothorax. The usual displacement of the heart, especially in artificial pneumothorax where the internal more than counterbalances the atmospheric pressure, strange to say, is accompanied by few signs of distress, or by no serious results. Of course, left-sided pneumothorax causes the greater displacement. While it might be expected, disturbance of circulation is by no means constant, even with decided displacement of the heart. Displacement downward of the diaphragm, the liver, and other abdominal organs, of course, would be a more or less natural result, according to the volume of the pneumothorax.

Among the most common accidental results of artificial pneumothorax may be mentioned emphysema. This condition is usually of short duration and apparently of minor importance. It appears to be the result largely of faulty technique, and I believe should properly rank with the more infrequent accidental complications. The injection of the air into the chest wall or into the lung substance rather than intra-pleurally, or its too voluminous introduction into the pleural cavity, are the most frequent causes of this condition. Defective apparatus might be responsible for this result.

Hydrothorax, which usually accompanies accidental, and not infrequently, artificial pneumothorax, is one of the complications that deserves close attention on account of the fact that it is possible for the effusion to assume massive proportions, thereby endangering the safety of the patient. But fortunately it is frequently in the nature of an aid and helps to more firmly fix and maintain the desired compression of the lung.

Pulmonary apoplexy is fortunately among the rare accidents, but holds a place of primary importance on account of real danger and alarming symptoms. I venture to suggest that faulty technique and the rupturing of pulmonary aneurisms are largely responsible for this accident. It is well known that aneurism of the lung is a very common result of pulmonary phthisis.

The most serious accidental result recorded is the production of hemiplegia. This most peculiar condition has been explained in different ways, the most probable theories being, 1st, the reflex theory, and 2nd, the gas embolism theory. Janeway reports two attacks of hemiplegia in the same case, resulting from the use of hydrogen dioxide in a sacculated pleural empyema. He also notes a case published by Laudet where hemiplegia followed the irrigation of an empyema cavity with a solution of iodine. The gas embolism theory seems to be the logical one, and faulty technique, therefore, probably plays quite an important role.

Puncturing of a blood vessel could result in gas embolism and also cause more or less hemorrhage. Puncturing of the lung substance, or of a bronchus, would result in more or less hemorrhage, and the injection of the gas into the respiratory tract. Of course, in this case, there would be no production or pneumothorax, as the gas would escape through the natural channels.

Infection, being clearly the consequence of improper sterilization, and naturally resulting in more or less empyema, or possibly in some other complication, is comparatively rare. When empyema follows as has been shown, it is not to be taken too seriously.

Failure to inject gas into the pleuritic cavity would be due to pleuritic adhesions, faulty technique, or improper apparatus. A small amount of gas might be injected into a pocket cut off by pleuritic adhesions. A number of such cavities could be injected, and by this means a more or less perfect pneumothorax produced.

### The Diagnosis of Pneumothorax.

The diagnosis of pneumothorax is important in the artificial method on account of the necessity of knowing when a sufficient volume of gas has been injected, and when it should be reinforced. The injected side is immobile and distended. The heart is more or less displaced. The vocal fremitus is absent or diminished. The resonance may be tympanetic, or even amphoric, skodiac, or possibly actually dull. The breath sounds are feeble or entirely suppressed, according to the amount of pressure. Rales, when heard at all, have a metallic quality. There may be the tinkling of Laennec. This peculiar metallic quality is also imparted to the voice. The coin-sound or the bruit-d'airain of Trousseau, is quite characteristic. When there is effusion the Hippocratic succussion is present. And lastly, the X-ray furnishes the most complete diagnostic method we have. With the used of a good X-ray and radiographic outfit the condition can be kept up with perfectly.

### The Operation.

Nitrogen has been chosen as the ideal agent for the production of pneumothorax, on account of its chemical inertness, and being slow of absorption. Unverrich demonstrated by means of chemical analyses of the air injected into the pleural cavities of dogs that the different gases are absorbed with varying degrees of rapidity.

Oxygen disappeared first, then carbon-dioxide, and at last the nitrogen. In the first case reported by Murphey, it was demonstrated by repeated physical examinations and by radiography that the lung remained collapsed something over two months. In other cases reported at the same time the absorption of the gas took place somewhat more rapidly. Lemke's experience is that the absorption had progressed sufficiently to make the breath sounds audible at the end of three and a half or four weeks.

Forlanini reports one case where the pneumothorax had been maintained for two years, which persisted for two additional years after the cessation of the injections.

According to Lemke: "From clinical observation in cases of natural as well as artificial pneumothorax, it is evident that the rate of absorption depends, aside from the chemical composition of the gas, on the state of the serous membrane and on the pressure which the gas exerts upon it. Hydrothorax may disappear rapidly or very slowly, depending on the condition of the pleura and the circulatory system. In simple serous effusions the point is reached when the pressure on the pleura becomes so great that it would seem absorption were practically impossible, and it is a well-known clinical fact that after a removal of a comparatively small amount of fluid absorption may go on quite rapidly. This has been accounted for by the supposition that the relief of the immense pressure permits of the opening of the points of absorption. The gas may be absorbed in much the same way and it is possible that the condition of the pleura and degree of distension of the cavity may govern the rate of absorption."

Now in massive injections there is no question about the almost imperative necessity of the use of sterilized nitrogen for obtaining the best and most permanent results. As this method of producing a complete pneumothorax at one injection has been abandoned for good and sufficient reasons in favor of the gradual production by multiple injections, there is no reason why sterilized air should not be employed for a time at least, with the final use of nitrogen to fix more firmly the condition. As air is approximately by volume 20.9% oxygen and 79.1% nitrogen, we would have this proportion of nitrogen left, as the oxygen would be absorbed quite rapidly. This would be no disadvantage, as the absorbed oxygen would tend in a slight degree to counterbalance the oxygen cut off by the compression of the lung, and would lead to a quick relief from over-distension. This method has been quite recently used successfully.

The length of time necessary to keep the lung immobile in order to cure would vary somewhat according to circumstances. The remarkable results obtained by Murphey and Lemke with one massive injection, in some instances repeated a few times, show the amazing efficiency of pneumothorax. It seems that the original idea of Murphey was to merely put the lung at rest with one, or a few treatments, and to let the case go at that. Not so with Forlanini. His system not only contemplates immobility of the lung, but to get the benefit of more or less strong compression, and to maintain this condition by any required number of injections to the point where the whole tubercular picture has been entirely replaced by that of pneumothorax. He suggests the indefinite maintenance of the pneumothorax not only as a cure of the diseased lung, but as a protection to the other lung, and after a cure of the phthisis has been accomplished, for the purpose of preventing the deformity of the thorax and functional disturbances and dislocation of adjacent organs, which would follow with a carnified lung and absorption of the gas in the pleural cavity. The term indefinite is used in a relative sense, as pneumothorax may become permanent, for reasons not absolutely understood, but likely due to a carnified lung in which no counter-pressure could be exerted, and on account of permanent relaxation of tissues producing no opposing influence, and, lastly, and most likely, because of a thickened pleura, in which the avenues of absorption have been entirely closed, this condition of permanent pneumothorax is accomplished. An absolute, perfect, indefinite continuation of the condition may not be altogether desirable on account of the fact (as has been demonstrated), that it is possible for the normal portions of the lung to resume its functions after obliteration of the tuberculous processes, thus complying with the conditions most desired, as suggested by Tidey.

As to what cases are suitable for the application of this method, Lemke answers, "that whereas bilateral are not as favorable as unilateral cases no matter what treatment is instituted, yet compression is not contra-indicated of the lung more advanced in the disease." Forlanini's experience leads him to assert: "The precise indications are monolateral lesions of considerable extent with non-adherent pleura, no matter whether there is a lesion in the other lung or not, provided it is in the initial stage."

### Results.

In 68 cases which I have analyzed, I find the following conditions before and after the operations:

53 Lemke, 8 Fontana, 5 Forlanina, 2 Orlandi.

The average ages of 65 of these cases were 28 years, 10 mo., the youngest being 14 and the oldest 60.

Of the 66 whose sex was given, 38 were male and 28 were female; of the latter 12 had married, one of whom was reported to have gone through a successful pregnancy after the establishment of the cure, which had no bad after effects.

The average gain in weight in 38 cases where the amount was reported was 8¾ lbs. Of the 68 cases, 49 were reported to have gained weight, two remained stationary, and 2 lost in weight, one of these was overweight at the beginning of the injections. Sixteen gained 10 pounds or more, 7 gained 15 pounds or more, 4 gained 20 pounds or more, the greatest gain being 28 pounds. It must be remembered in this connection the large majority of the cases were under observation a comparatively short time.

Of 46 cases the average time under treatment was approximately three months. Four additional cases were under treatment and observation, one for one year, one for one year and three months, one for three years, and one for five years. Quite a number of cases were lost sight of in a short time.

It is interesting to note that of 49 cases where family histories were given, 32 were negative and only 17 positive of tuberculosis.

Of 61 cases where the amount of sputum was given before operation, it was profuse in 30, free in 6, moderate in 23 and slight in 2.

After operation sputum was entirely absent in 23 cases, in 15 it was very slight, usually amounting to a little in the mornings, in 7 slight, and 10 diminished.

The cough after operation was entirely absent in 22 cases, very slight in 11, slight in 7 and diminished in 14. In the cases where tubercle bacilli were reported to have been found they were present in 39 cases before operation and afterwards entirely absent in 21 cases, very few in 3 cases, and diminshed in 1 case.

There were a number of cases where expectoration had entirely ceased, where no report was made of a search, but, of course, the inference is that the tubercle bacilli were absent, and the supposition is that they were present in all cases in the beginning, as these reports were made by very competent observers.

The mean temperature in 56 cases before operation was 101.1 and the average of 48 in the final result after operation was 98.9. The average pulse rate in 44 cases was 109 before operation, and afterwards 95 in 36 cases, the others not reported. In 22 cases the rate of respiration was 24.1 before and 23.4 after.

Following operation there was empyema in 4, pain in 9, one case of air embolism, the needle entered the lung tissue in 3 cases and air was injected into the bronchi twice. Five cases developed hydrothorax after operation, and 1 empyema; 18 cases had dyspnoea, 1 pulmonary apoplexy, and 2 syncope.

The final results were perfect in 17 cases, good in 33, improved in 8, slightly improved in 2, arrested in 1, negative in 2, bad in 1. There was failure to inject in 1 case, 1 died (not as a result of operation), and 2 were lost sight of.

In considering the final result it must be remembered that 53 were cases of Lemke's in which one or a few massive injections only were given, and practically all of them were under observation for a short time only. Of the remaining 15 cases, 2 reported by Orlandi, 8 by Fontana, and 5 by Forlanini, where multiple injections were given and the processes maintained, the results were perfect in 13 (86.6%), the progess arrested in 1, very good in 1. These last results give a correct idea of what may be expected of the proper application of artificial pneumothorax.

The question arises as to what we can attribute the remarkable results obtained on all hands by the practice of artificial pneumothorax. There is beyond doubt a subjective and clinical recovery, a complete cure, obtained from the correct application of this method. We readily see that we have an immobilized lung. The great therapeutic aid of rest is here obtained most perfectly. All cavities and diseased portions of the lungs are emptied of their foul contents. Here the great surgical aid of drainage is carried out most completely, with consequent stoppage of absorption of the poisonous products of tubercular as well as mixed infection, which is always concomitant. There is co-aptation of the walls of the cavities and of ulcerated surfaces, thus complying with a well-known surgical principle necessary for healing.

The consequent edema resulting from pressure very likely has its influence in a curative way. This idea has support in the results obtained from the well-known Bier method of treatment. It might be possible that pressure lessens or prevents the absorption of toxins. On the other hand, it might also be the case that certain substances are forced into the system by pressure, which result in the formation of antitoxic bodies. The results obtained by the tuberculin treatment would lend color to this theory. Murphey says that a favorable influence exerted by a compression is due to the mechanical obstruction of the various avenues by which this infection is transmitted to other parts of the lung, the most important avenues being the bronchi, the air vescicles and lymphatic circulation.

Forlanina observes, "that pneumothorax cures the destructive processes of phthisis by rendering the lung absolutely immobile; heals the solution of continuity already formed, compresses cavities, empties them, and by the coalition of their walls brings about their obliteration."

Now, since it has been shown that pneumothorax properly applied and maintained is positively curative of tuberculosis of the lung, why should this method be withheld from any case possible for its application? The proportion of cases where the method can be employed would include practically all those of monolateral tuberculosis, except where the pleura is too adherent. It has been proven that a tuberculosis of a mild grade in the opposite lung from the one most affected is very favorably influenced by a production of pneumothorax in the more diseased side. According to Lemke, the earlier treatment is begun the better the prognosis. Then why should not this treatment be instituted as soon as the disease is discovered, whatever the condition, provided the adhesions are not too extensive, and the disease is not advanced too far in the opposite lung? The fact that the tuberculosis is in the initial stage, or of a mild grade, would be no excuse for withholding this, a positive curative method, no more so than we would be justified in failing to apply a curative remedy or surgical operation when indicated in other diseases.

Other aids in the treatment of pulmonary tuberculosis, as tuberculin, sanatoria, climatic, and dietetic methods, would not be by any means precluded in the application of pneumothorax, They would of course be of immense aid in some cases, or possibly all. But one of the strong arguments in favor of this method is that cures are accomplished by it alone.

Three patients whom I operated on in August of this year in connection with Dr. Mary E. Lapham, were injected in the beginning with sterile air. I have recently heard from two of these cases, and in both the results are very gratifying, one having resumed his ordinary occupation. These three cases will be reported in detail at a later date.

This analysis of artificial pneumothorax deals entirely with the results obtained from the intra-pleural method of injecting air or nitrogen. The lung is also collapsed and held practically immoble by the operation of Freidrich and Bauer, known as *complete pleuro-pneumolisis.* Very brilliant effects have been obtained by this operation, and the final result in the lung being very similar to those following pneumothorax, although accomplished in a different manner. Of other material than air or nitrogen, a weak aqueous solution of oil of cloves has been used more or less successfuly in one case. Loomis experimented with sterilized plaster of Paris, cocoa-butter, etc., but his attempts met with failure.

